# ISG15–LFA1 interactions in latent HIV clearance: mechanistic implications in designing antiviral therapies

**DOI:** 10.3389/fcell.2024.1497964

**Published:** 2024-12-24

**Authors:** Prasad S. Koka, Bharathi Ramdass

**Affiliations:** Biomedical Research Institute of Southern California, Oceanside, CA, United States

**Keywords:** interferon types-I/II, interferon stimulated gene-15, lymphocyte function-associated antigen-1, endothelial stem-progenitor cells, hematopoietic stem-progenitor cells, naïve/resting activated cell–cell contact, intercellular HIV transfer, HIV latency reversal

## Abstract

Interferon types-I/II (IFN-αβ/γ) secretions are well-established antiviral host defenses. The human immunodeficiency virus (HIV) particles are known to prevail following targeted cellular interferon secretion. CD4^+^ T-lymphocytes are the primary receptor targets for HIV entry, but the virus has been observed to hide (be latent) successfully in these cells through an alternate entry route via interactions with LFA1. HIV facilitates its post-entry latency-driven mode of hiding through these interactions to displace or inhibit ISG15 by forming the HIV1-LFA1 complex *in lieu* of ISG15-LFA1, which would at least transiently halt and bypass type-I IFN secretion. This could explain why the elimination of HIV from cellular hideouts is difficult. Hence, HIV clearance needs to be addressed to reverse its latency in LFA1^+^ T-lymphocytes and CD34^+^/CD133^+^ early progenitor stem cells. In the context of hematopoietic or endothelial stem-progenitor cells (HSPC/ESPC), we discuss the potential role of LFA1 in HIV permissiveness and latency in LFA1^-^CD34^+^/CD133^+^ versus LFA1^+^CD34^+^/CD133^+^ HSPCs/ESPCs. In HIV latency, the viral particles may remain engaged on the naïve-resting cells’ LFA1, which are then unable to accommodate the ISG15 molecules owing to conformational changes induced upon occupation by the virus at the ISG15-LFA1 binding or interaction sites through halting of the subsequent downstream type-II IFN secretion. Viral binding to LFA1, including its transfer through activated-naïve cell–cell contacts may be a key step that needs to be addressed to prevent “transient or partial” virus-induced shutdown of type-I IFN secretion. This process allows an alternate viral entry and hideout site via LFA1. The subsequent administration of recombinant ISG15 may ensure sufficient type I/II IFN release to promote, enhance, or sustain the innate immune responses. Thus, combination antiviral therapies could potentially include exogenous ISG15 to maintain or sustain biologically and clinically relevant ISG15-LFA1 interactions. In addition to alternating with co-challenges of PKC-pro-LRA-drug modulators, this is administered post (antiretroviral therapy) and continued with periodic ART until permanent elimination of viral resurgence and latency is achieved in patients with HIV/AIDS. This triple-combination drug regimen is expected to pave the path for systemic virus clearance *in vivo*.

## 1 Introduction

Viruses, including the human immunodeficiency virus (HIV)-1, often seem to find ways to overcome the host antiviral defenses of cellular interferon secretions (García-Sastre, 2017), such as in lymphocytic choriomeningitis virus (LCMV) infection (Perro et al., 2020). Conventional antiretroviral therapy (ART) regimens (Withers-Ward et al., 1997; Koka et al., 1998; Broder, 2010; Volberding and Deeks, 2010; Liner et al., 2010) for containing or eliminating HIV infection are inadequate for complete viral clearance in infected individuals. The primary cause of this viral elusiveness to ART is its ability to hide in the infected cells and escape immune surveillance. Investigative efforts to eliminate the viral particles from their cellular hideouts have shown increasing efficacies. These latency-reversing agents (LRAs) are being continuously “tailored” to achieve increasing risk-versus-benefit intended for HIV/AIDS patients in ongoing sequential experimental approaches using humanized mouse model systems (Brooks et al., 2003; Mehla et al., 2010; Sloane et al., 2020; Marsden et al., 2020; Chen et al., 2022; Dimapasoc et al., 2024; Ngo et al., 2024).

Although CD4^+^ T-lymphocytes are the primary receptor targets for HIV entry that ultimately lead to productive infection, the virus has been shown to enter these targeted CD4^+^ T-cells through an alternate route via interactions with the lymphocyte function-associated antigen (LFA)-1 (Hioe et al., 2001; Beauséjour and Tremblay, 2004; Tardif et al., 2009; Kondo and Melikyan, 2012). Complexation of the virus with LFA1 suggestively impedes the interferon stimulated gene (ISG)-15 from attaching to LFA1, resulting in at least transient prevention of type-I IFN secretion (Boasso, 2013; Swaim et al., 2017). This facilitates the virus as an alternate route for its latency-producing entry and a mode to hide primarily in naïve or resting T-cells or in early progenitor stem cells, following which the virus also shuts-down, impedes, or interferes with type-II IFN secretion (Swaim et al., 2017). Otherwise, if not blocked by the virus, the human host can defensively counteract the pathogen invasion by type-II IFN-mediated killing of the productively HIV-infected cells along with simultaneous elimination of the virus particles by host reaction-elicited effector cell vesicles (Perng and Lenschow, 2018; Fernández et al., 2020; Kespohl et al., 2020).

IFN secretions are influenced by ISG15-LFA1 interactions that elicit innate immune responses through cytotoxic T-lymphocyte (CTL) and natural killer (NK) effector cells. Modified Vaccinia virus Ankara (MVA)-based recombinant vectors that express HIV1 Env/Gag-Pol-Nef and ISG15 show that the Armenian hamster ISG15 overexpression can increase type-I IFN production and enhance HIV-specific immune responses, specifically by enhancement of the HIV-restricted CTLs in immunized rodents (Falqui et al., 2023; Gómez et al., 2020). These observations are attributable to the difference in the ISGylation function of the mutant ISG15 (negatively) compared to wild-type ISG15 (positively) and thus to the ISGylation-dependent activation of LFA1. Other studies in mice unrelated to HIV have shown that CTL responses were enhanced in consequence to the initial ISG15 influences on NK cells (Iglesias-Guimarais et al., 2020; Villarreal et al., 2015). This NK-CTL functional relationship is relevant for its occurrence or dependency in the context of ISG15-delivered innate immune responses. However, species-specific molecular structures and ISGylation vis-à-vis immune function differences in ISG15 may exhibit variable relevancy or degree of innate immune response efficacy (Speer et al., 2016; Krishna Priya et al., 2022). Extending the reach of this efficacy may be inadequate to eliminate HIV from its latency hideouts since the virus may seek refuge in LFA1 (the receptor of ISG15) to escape immune surveillance.

Entry through LFA1 could be the reason why HIV is difficult to be eliminated from its host cellular hideout, and immune suppression or evasion is extended through the naïve or resting host CD4^+^ T-cells (Platanias, 2005; Wang et al., 2009; Starling and Jolly, 2016; Kondo et al., 2022; Shi and Shao, 2023). Thus, the latent HIV must be cleared from LFA1-mediated binding to T-lymphocytes ( Han et al., 2004; Dai et al., 2009; Ramakrishnan et al., 2012; Wietgrefe et al., 2023) as well as from the virus-entry-permissive early-differentiation-stage hematopoietic CD34^+^ or CD133^+^ endothelial stem-progenitor cells (HSPCs/ESPCs) for total virus clearance (McNamara et al., 2012, 2013).

We previously reported that these HSPCs are resistant to productive direct HIV1 infection *in vivo*, and activation of viral presence in the HSPCs results in their imminent apoptosis (Koka et al., 1999; Padmanabhan et al., 2020; Koka and Ramdass, 2023, 2024). In the context of these HSPCs or ESPCs, we also discuss the relevance of LFA1 in potential HIV permissiveness and latency in LFA1^-^CD34^+^/CD133^+^ versus LFA1^+^CD34^+^/CD133^+^ HSPCs/ESPCs.

Cellular secretion of ISG15 from T-lymphocytes and certain other cell types signal LFA1 in both an autocrine or a paracrine manner (Swaim et al., 2020). We propose the blocking of viral binding to LFA1 through restoration of ISG15-LFA1 complexation as necessary until permanent viral clearance as a key step to preventing viral interference in IFN secretions. In this context, administration of exogenous recombinant ISG15 may ensure type-II IFN (Zhao et al., 2005) and perforin (Spaner et al., 1998; Kaser et al., 1999; Hersperger et al., 2010) secretions of lytic granules release by the CTL and NK cells in innate immunity responses. Thus, we propose that combination antiviral therapies include exogenous recombinant ISG15 (Bogunovic et al., 2013) administration post-ART to achieve minimal undetectable viral loads, along with continued and periodically intermittent alternating ART challenges with the proposed protein kinase C (PKC)-pro-LRA-drug modulators (Brooks et al., 2003; Mehla et al., 2010; Sloane et al., 2020; Marsden et al., 2020; Chen et al., 2022; Dimapasoc et al., 2024; Ngo et al., 2024) for complete HIV clearance *in vivo*.

## 2 LFA1 as dual alternate cellular receptor for HIV1 entry and latency hideout

**FIGURE 1:**
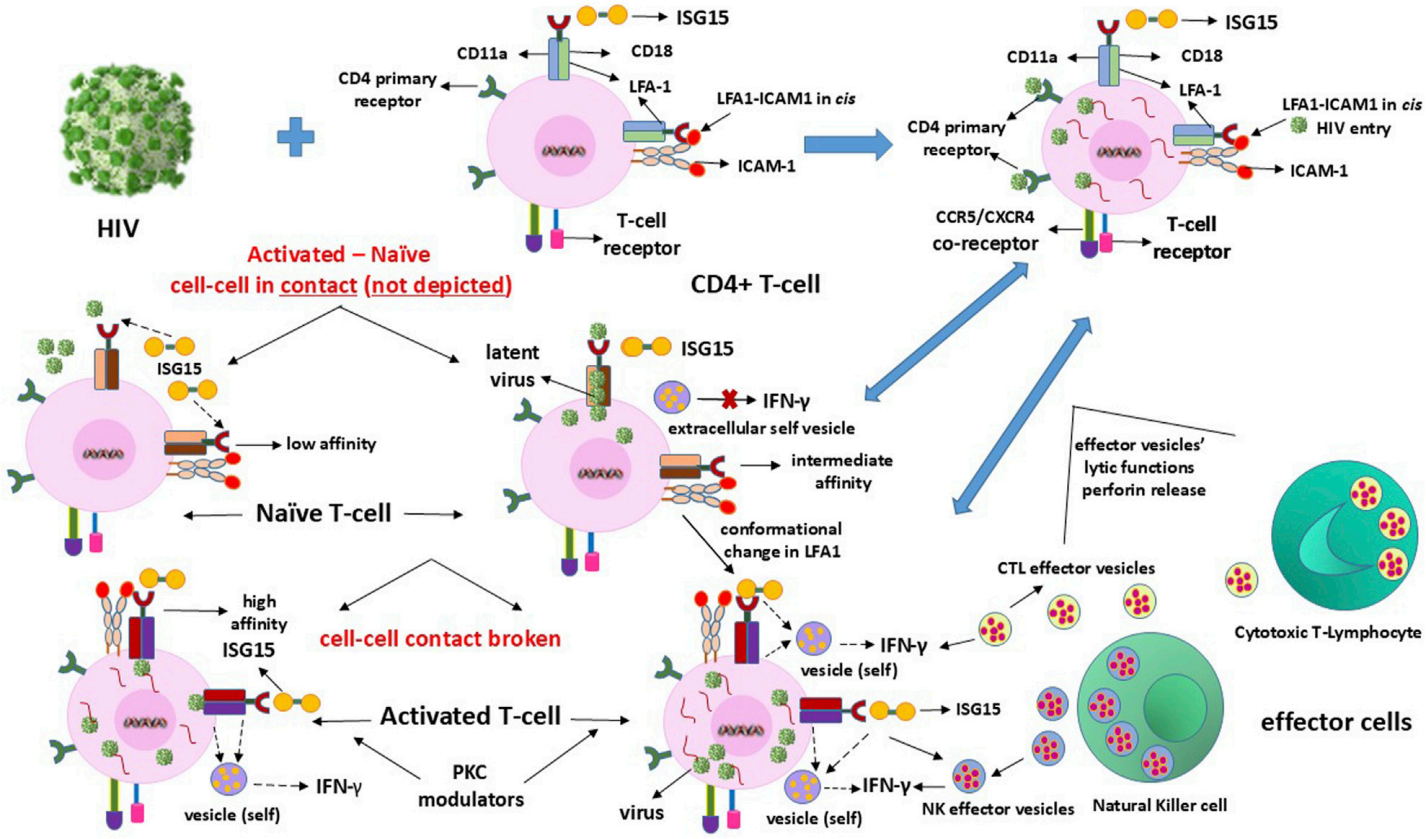
Mechanisms of clearance of HIV latency in or from CD4^+^ T-cells. The infection is ultimately eliminated by the IFN-γ-induced effector vesicles of the cytotoxic T-lymphocytes (CTLs) and natural killer (NK) cells that attack and kill the HIV-replicating cells.

HIV1 enters the T-cells through dual routes comprising the primary CD4 antigen and LFA1 interactions (Hioe et al., 2001). Viral entry using the receptor CD4 antigen ensures productive infection when the T-lymphocytes are activated (Wang et al., 2009). We postulate that the dual entry modes of the virus, which include LFA1 (Hioe et al., 2001; Beauséjour and Tremblay, 2004; Tardif et al., 2009; Kondo and Melikyan, 2012), provide the pathogen a “home” within the same CD4 phenotypic receptor cells to evade immune surveillance. However, latency may not be maintainable or sustainable when the entry occurs solely through the CD4 primary receptor of the virus when the cells are in an activated state instead of the naïve resting state. LFA1 in the “vacated” state unoccupied by ISG15 is seized by the virus to facilitate its latency-driven mode of entry, enabling escape or evasion of host immune surveillance by at least transient and partial prevention of the outside-in signaling. This expectedly occurs from the binding of type-I IFN to the interferon alpha receptor (IFNAR) on the cell surface (Platanias, 2005; Swaim et al., 2017). Consequently, downstream type-II IFN gamma secretion is aborted, which would otherwise initiate the IFN-γ-mediated release of extracellular vesicle granules to achieve perforin-mediated killing of the productively infected activated CD4^+^ cells (Spaner et al., 1998; Kaser et al., 1999; Hersperger et al., 2010) (Figure 1). This is possible owing to the triggering of the ISG15-induced innate immunity-directed vesicles released from the NK cells or by the CTLs for their lytic action on the virus-replicating CD4^+^ cells.

## 3 Expected LFA1 conformational changes and cell activation state facilitating HIV entry and latency

**FIGURE 2:**
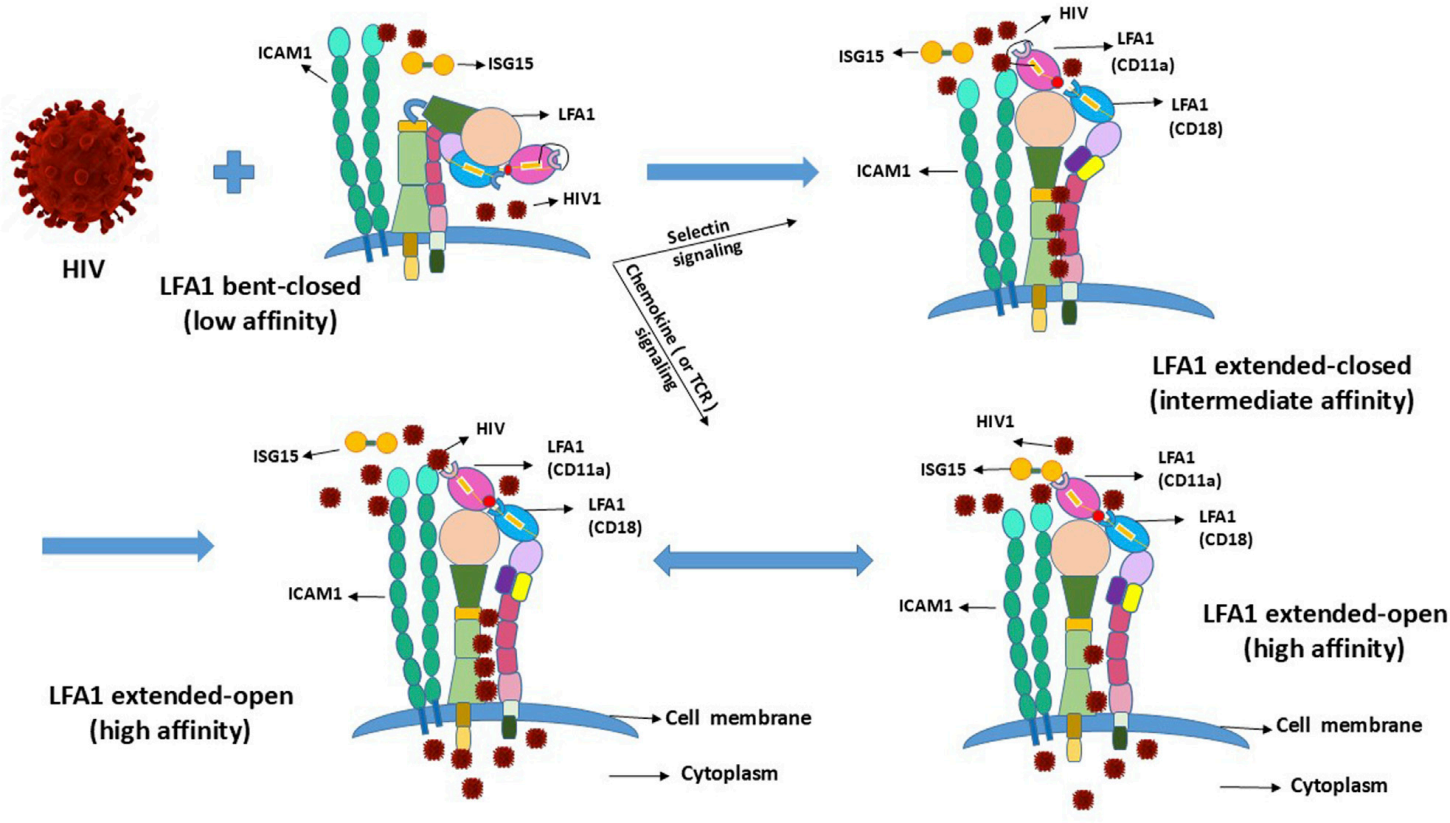
Conformational changes of LFA1 due to outside-in and inside-out signaling and their projected influences upon interactions with HIV.

The coupled dual-strand CD11a/CD18 integrin LFA1 exists in multiple conformations depending on the cellular naïve resting and activation states (Wang et al., 2009). LFA1 has three different conformations depending on the activation state of the CD4^+^ T-cells (Kondo et al., 2022). Activated T-cells require LFA1 to attain or transition into the high-affinity extended-open conformation following transition from the naïve resting state, where the inactive conformational state of LFA1 exists in a low-affinity bent-closed or an intermediate-affinity extended-closed conformation (Wang et al., 2009; Kondo et al., 2022; Shi and Shao, 2023). Expectedly, this cellular activation is incidental to parallel LFA1 activation, or vice versa to the naïve or resting states (Wang et al., 2009; Kondo et al., 2022). LFA1-activation-related conformational changes also involve cell-surface molecular interactions between the ligand, intercellular adhesion molecule-1 (ICAM1 or CD54) involved in leukocyte adhesion, and its receptor LFA1 (α_L_β_2_ or CD11a-CD18) (Long, 2011). Coincidental to the conformational changes of LFA1, the involvement of ICAM1 and its interactions with both the CD11a and CD18 strands of LFA1 presumably promote the latency-driven intrusions by the viral particles (Figure 2). Such facilitation of HIV entry can occur independent of LFA1 when the virus latches onto CD4 or is anchored onto the LFA1-ICAM1 complex from cell–cell contacts, thereby stalling the ISG15 binding to LFA1 that would otherwise signal IFN secretions to promote antiviral innate immune responses.

## 4 Importance of cell–cell contacts in subsequent latency-driven HIV passage from activated to naïve or resting cells in the stromal microenvironment *in vivo*

Intercellular contacts within the stromal microenvironment are essential for transferring the virus from the infected and activated cells harboring replicating viruses to naïve or resting cells to facilitate induction and maintenance of latency (Evans et al., 2010; Bracq et al., 2018; Walling and Kim, 2018). We postulate that this transfer may occur through LFA1 when the naïve resting cells begin to transition from a low-affinity bent-closed conformation to an intermediate-affinity extended-closed LFA1 conformation, preempting the ISG15 from occupying the different yet vacant integrin sites or domains for virus transfer or docking. Thereafter, during the resting–activated cell–cell contacts, both the cells and LFA1 are transitioned into their activated states as an interdependent requirement. The dual-activated high-affinity cells and LFA1 facilitate the docking of the virus particles onto the latency-supporting naïve resting T-cells, thereby blocking ISG15-mediated LFA1 interactions and outside-in signaling that would otherwise promote IFN-γ secretion required for antiviral activity. This supports the assumption that the virus can pass from the activated to naïve resting CD4^+^ T-cells, which may even have greater plausibility of occurrence during the activated–naïve intercellular contact *in vivo*.

Likewise, it is plausible that intercellular contacts between two resting CD4^+^CD25^−^ cells could induce LFA1 conformational changes so as to transfer HIV between these cells, with the caveat that a productive viral infection may occur in the resting CD4^+^CD25^−^ cells (Wietgrefe et al., 2023). This virus transfer is reportedly due to the presence of a functional positive transcription elongation factor b (P-TEFb) (Wietgrefe et al., 2023); P-TEFb is composed of cyclins T1 or T2 and cyclin-dependent kinase 9 that control or regulate the elongation phase of transcription by RNA polymerase II (Fujinaga et al., 2023). This helps the potentially smaller HIV transcripts become susceptible to full-fledged transcription of the latent or truncated infectivity-prone virus particles. The reported findings (Wietgrefe et al., 2023) lend credence to our postulate herein that the resting/naïve–activated intercellular contact is a prerequisite for HIV transfer across cells, unless the circulating plasma virus particles directly enter the primary cells via CD4, yet implausible via LFA1. However, such an argument may not be sustainable. Should a non-LFA1-driven CD4 virus entry mode from the activated to naïve/resting T-cells occur during cell–cell contact, immediate latency may not be prevented. However, viral replication is expected to resume post-activation of the virus-hosting resting/naïve cells. In this context, HIV entry via CD4 elicits ISG15-mediated innate immune responses and susceptibility to ART to attain “undetectable” virus loads, whereas virus entry via LFA1 halts or interrupts ISG15 availability for targeted pathogen killing and requires the action of LRAs. Furthermore, exogenous ISG15 supply to compensate for the potentially evident exhaustion of the CTL and NK effector cell potency levels is a necessary consideration (Table 1).

**TABLE 1:** Projected combination antiretroviral therapy (ART) regimen for complete systemic eradication of HIV infection in patients. The efficacy of the proposed inclusion of recombinant ISG15 with PKC modulators (PKCM) to release the latent virus and attack the pathogen through immune responses simultaneously is reflected in the IFN-γ levels. Furthermore, alternating cycles of ISG15+PKCM treatment with ART to nullify viral replication should lead to eventual viral clearance even beyond the latent minimal undetectable viral load to zero non-recurring infection level so that further LRA-PKCM is not required.

Event sequence	Therapy agent	Viral load	IFN-ƴ level	Infection status
1	ART	Minimal	Subnormal Suboptimal	Latent
2	PKCM + ISG15	Increase	Increase	Partial release + Latent
3	ART	Minimal	Subnormal Suboptimal	Neutralized + Latent
4	PKCM + ISG15	Increase	Increase	Partial release + Latent
5	ART	Minimal	Subnormal Suboptimal	Neutralized + Latent
6	PKCM + ISG15	Increase	Increase	Release complete
7	ART	Zero	Normal Optimal	Neutralized - Zero latency

## 5 Competition between HIV1 and ISG15 to occupy the LFA1–ICAM1 complex binding sites or domains

HIV1 may compete with the type-I IFN signaling of ISG15 to serve the dual purpose of its entry and hideout by utilizing the receptor CD4 (with CCR5/3 or CXCR4 coreceptors) (Koka et al., 1998; Zhang et al., 2010) and LFA1 (comprising CD11a+CD18 strands) engaged on the cell surface to replicate and hide, respectively (Wang et al., 2009; Kondo et al., 2022; Shi and Shao, 2023). This implies that these naïve resting and activated T-cells carry LFA1 in a particular conformation out of its three different states classified as low (bent-closed), intermediate (extended-closed), and high (extended-open) affinities (Wang et al., 2009; Kondo et al., 2022). This asks the question of which of these LFA1 conformations facilitates viral binding for entry and hiding. The virus presumably has to “lock-in” or “latch-on” to LFA1 when the head of this integrin molecule is available. This suggests intermediate- or high-affinity extended-head integrin conformation as suitable for initiating virus–LFA1 interactions but not the low-affinity LFA1 with the presumptive unavailable extracellular head (or domain) in the bent-closed conformation (Wang et al., 2009; Kondo et al., 2022) (Figure 2). Some differing reports exist in that the LFA1 ligand ICAM1 may play a role in the cell–virus interactions in a stabilizing capacity (Tardif and Tremblay, 2003; Kondo and Melikyan, 2012) that may favor latency or syncytium formation (Yu et al., 2020) where the replicative virus particles are released into the intercellular environment. Cells involved in syncytia-induced fusions may exist in the activated state of LFA1, promoting replication of the released virus and thereby eliciting ISG15-mediated innate immune responses. Syncytia-induced fusions that do not deliver latency are expectedly different from the resting–activated cell–cell contacts described herein that induce virus latency. Cell fusions of syncytia formation are more possible for the CD4^+^ T-cells upon infection by the CXCR4-tropic HIV strains but not for cells that do not support productive infection of the replicating virus particles, such as the stem-progenitor cells (Koka et al., 1998; Koka et al., 1999; Zhang et al., 2010).

## 6 Permissiveness of HIV in the productive-infection-resistant CD34^+^/CD133^+^ stem-progenitor cells

**FIGURE 3:**
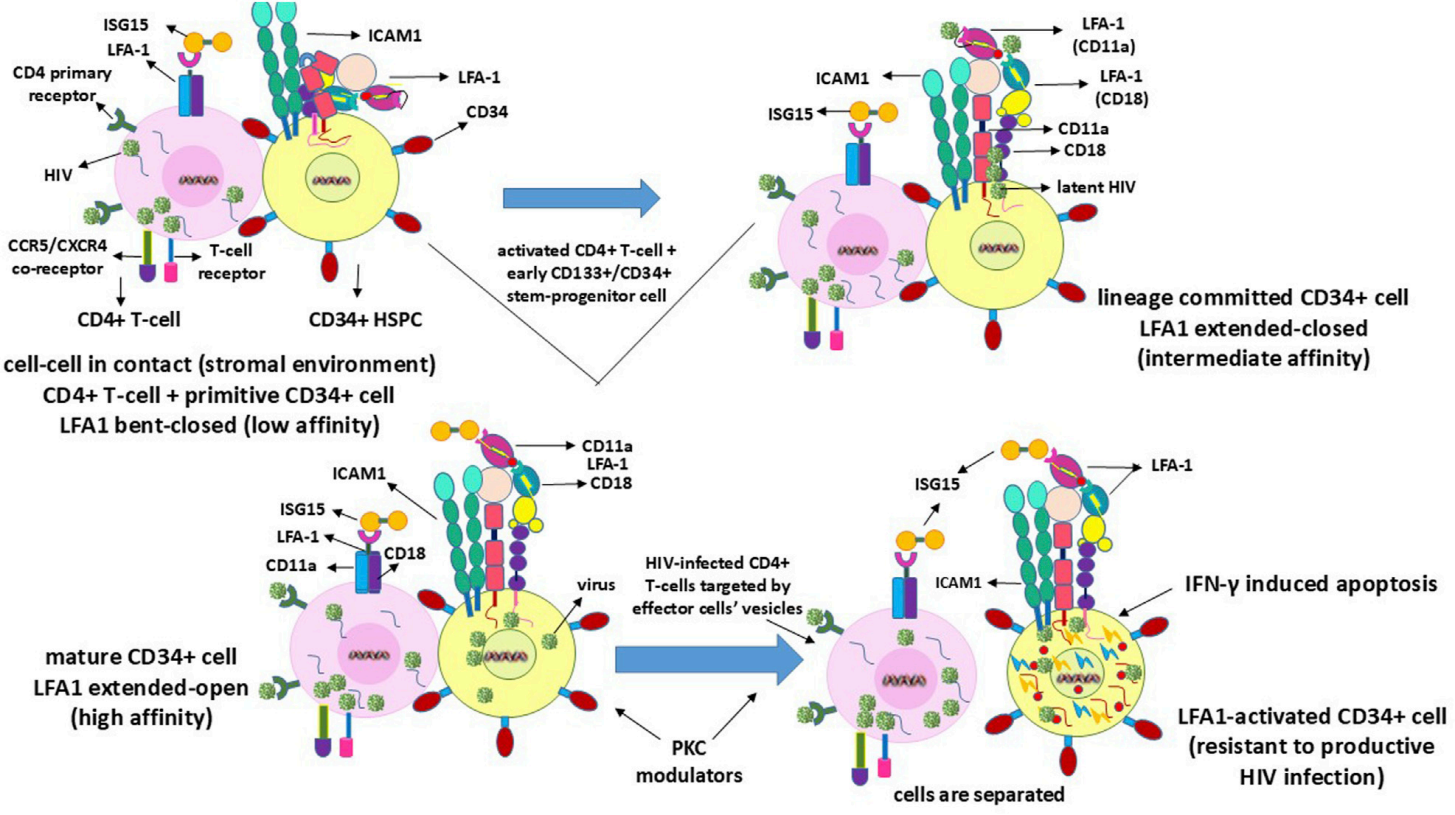
Mechanisms of clearance of HIV latency in or from CD34^+^ or CD133^+^ stem-progenitor cells. Since these cells do not sustain a productive HIV infection, latency reversal within the cells ultimately terminates through IFN-γ-induced apoptotic self-lysis.

HIV latency in the stem-progenitor cells was reported previously (McNamara et al., 2012, 2013). In this regard, the purportedly implicated CD34^+^ HSPCs or CD133^+^ ESPCs (Gunji et al., 1992; Torensma et al., 1996) do not survive and presumably undergo rapid apoptosis upon viral entry or transfer into these cells (Koka et al., 1999; Zeng et al., 2006), as depicted in Figure 3. Hence, the virus is expected to enter the HSPCs/ESPCs via LFA1 as the early or primitive progenitor LFA1^-^CD34^+^/CD133^+^ (or even LFA1^-^CD34^-^/CD133^+^) cells differentiate into the LFA1^+^CD34^+^/CD133^+^ cells even as the “silent-intrinsic” lineage commitment is not expressed phenotypically (Gunji et al., 1992; Torensma et al., 1996). This further suggests that these cells may not have acquired the CD4^+^ T-cell lineage commitment without explicit expression of this phenotype, an argument that supports the reported virus latency in these stem-progenitor cells independent of P-TEFb (McNamara et al., 2012, 2013). The cellular receptor scenarios for virus entry are different in the CD34^+^ HSPCs and CD133^+^ ESPCs than in the CD4^+^ T-cells, which comprise both the viral primary cellular receptor CD4 antigen and alternate latency-driven secondary LFA1 integrin. However, LFA1 on the non-committed stem-progenitor cells, despite expressing only a coreceptor CXCR4 or CCR5 for virus entry, may also provide a refuge for the virus in the latency-driven hideout mechanism. The actual mode of latency-driven virus entry from the activated T-cells into naïve resting T-cells or into CD4 receptor-lacking HSPCs or ESPCs through LFA1 may or may not be different. The primary structural differences at the amino-acid level between different LFA1 molecules are expected to be remote even though the cell types involved in intercellular contacts are not the same. Furthermore, stem-progenitor cells are replete with multiple differentiation stages that are not possessed by terminally differentiated T-cells. Hence, differences between these cell types, including the expression levels of various transcription factors of the CD34^+^ stem-progenitor cells (Kim et al., 2009; Bai et al., 2010; Gomes et al., 2002; Hughes et al., 2020; Sonoda, 2021), can influence cell fate, lineage commitment, or differentiation stage at the onset of viral entry and latency deliverance. Moreover, transcription factors other than P-TEFb may be involved in the susceptibility to shelter latency. These occurrences, in turn, have consequences on the susceptibility of the stem-progenitor cells to ISG15-LFA1-mediated antiviral activity. This is because these cells do not harbor a productive HIV1 infection but are expected to undergo rapid apoptosis upon any intracellular virus release even prior to CTL or NK cell effector activity. Herein, LRA action on the stem-progenitor cells is expected to enable latent virus release, such that the cycles of ART and ISG15+PKCM may be utilized as proposed (Table 1).

## 7 Different events related to cell–cell contact for LFA1-mediated HIV entry into the stem-progenitor cells

Although naïve resting T-cells already express the CD4 virus receptor CD4^+^CD25^−^ phenotype, the CD34^+^/CD133^+^ early or primitive stem-progenitor cells do not have such a primary cellular mechanistic entry route even when the virus coreceptors CCR5 or CXCR4 have been implicated (Carter et al., 2010, 2011; Sebastian et al., 2017; Zaikos et al., 2018). These cells exist in multiple stages of differentiation, including primitive, lineage-committed, mature, and activated states, with LFA1 in the low-affinity bent-closed, intermediate-affinity extended-closed, high-affinity extended-open, and potentially apoptotic correlating roles (Gunji et al., 1992; Torensma et al., 1996; Zeng et al., 2006), similar to the T-cells but with exception of apoptotic events from intracellular mechanistic conditions (Wang et al., 2009; Kondo et al., 2022). Hence, the virus could have a time-delayed latency-driven entry into the stem-progenitor cells after coming into contact with the T-cells since the differentiation stages of these cells need to be considered (Gunji et al., 1992; Torensma et al., 1996). Whether a purported CD4-anchorage-independent virus entry into the progenitor cells is plausible, and if so the differentiation stage at which such entry can occur is further complicated by the occurrence of multiple differentiation stage phenotypes for cell lineage vis-à-vis virus permissiveness. The virus remains dormant or latent during these differentiation stages of the stem-progenitor cells before cell maturation, and simultaneous LFA1 activation leads to imminent cellular apoptosis (Figure 3). This mechanism is different from the activation of the effector function induced by the virus in the CD4^+^ T-cells, thereby releasing lytic granules (Figure 1). The reason for this variation in infected cell killing or death is that the replicative potential of the virus in the infected thymocytes potentiates an effector influence (Hersperger et al., 2010), whereas the inability of the virus to replicate in the CD34^+^ HSPCs or CD133^+^ ESPCs (Koka et al., 1999; Padmanabhan et al., 2020; Koka and Ramdass, 2023, 2024) elicits a suicidal apoptotic influence (Zeng et al., 2006). Sustained effector functions are important for virus clearance when innate immunity is elicited in the replicating mode through the influence of ISG15, but this is unlikely with regard to replication-resistant stem-progenitor cells where apoptosis is the likely event (Figure 3). Virus replication may resume when these cells acquire mature cell phenotypes or become LFA1 activated upon differentiation. In the unlikely event that the terminally differentiated stem-progenitor cells acquire a CD4^+^ T-cell phenotype prior to apoptosis, then the effector function may be invoked at that stage. However, LRA action may preempt apoptosis, at which time the cycles of viral release and killing by ART and ISG15 treatments emerge as necessary options (Table 1).

## 8 Transient or partial blocking of ISG15-mediated interferon secretions by the virus entry to perpetuate pathogen survival and latency

The usurpation of LFA1 sites by HIV may effectively block the legitimate binding of ISG15 or ICAM1 to the integrin and promote pathogen entry (Figure 2) by downregulating both type-I IFN and subsequent downstream end type-II IFN secretions that block normal outside-in signaling mechanisms (Figure 1). These antiviral responses are requisites in their intrinsic innate immune responses and effector functions of human host cells *in vivo*. The most resistive influence of HIV is two-pronged: first, the virus evades humoral antiviral immunity through excessive and rapid multiple replicative strain elicitation potential; second, the virus has conceived or envisaged an efficient latency mechanism to hide from the CTL and NK cell effector functions by entering through LFA1. ISG15 binding to LFA1 is necessary for inducing the innate immunity of the effector CTL and NK cells to kill the infected cells. Hence, an efficient type-I/II IFN-α/β/γ secretion process needs to be sustained for virus clearance, for which the binding of ISG15 to LFA1 is required or the requisite ISG15–LFA1 interactions must be maintained.

## 9 Recombinant ISG15 inclusion as part of combination therapy for virus clearance

Complementation of the exogenous recombinant ISG15 post-ART induced undetectable viral loads in the human host, together with the PKC-agonist LRAs (Tanaka et al., 2022; Grau-Expósito et al., 2019; Ait-Ammar et al., 2020; Spivak and Planelles, 2018; Rodari et al., 2021; Debrabander et al., 2023) is worthy of investigation as proposed (Table 1). Periodically repeated treatments with these PKC modulators (PKCMs) (Fine et al., 1996; Lim et al., 2015) can help with PKCM-induced latent virus release for concomitant administration of exogenous recombinant ISG15 (Table 1). Intermittent or periodic recombinant ISG15 in combination with the LRA-prodrug PKCMs (e.g., bryostatin-1 or prostratin) (Sloane et al., 2020; Marsden et al., 2020; Dimapasoc et al., 2024) can be administered until complete viral clearance. Class-I selective histone deacetylase inhibitors were also suggested or utilized for HIV latency reversal in stem-progenitor cells (Painter et al., 2017; Zaikos et al., 2018). Since ISG15 reportedly blocks viral release late in the budding process (Pincetic et al., 2010), fully functional and efficacious LRA action may be achieved by alternating co-challenge of ISG15 + LRA-prodrug with ART in HIV-infected patients. A sequential PKCM immediately followed by ISG15 administration cannot be ruled out completely, but it may not prevent the virus from reinfecting the neighboring cells during the lag time without the suggested alternating treatment with ART. Furthermore, rejuvenation of impaired IFN-γ secretion by CTL and NK cells with exogenous ISG15 supply is an important consideration for preventing the exhaustion of these effector cells for maintaining lytic granule exocytosis and mediating perforin-induced killing of the virus-infected cells. Such a recharge potential may be achieved through periodic intermittent ISG15 challenges to sustain the IFN-γ induced exocytosis of the vesicles carrying the lytic granules, so that the spurts of LRA-prodrug mediated release of latent virus-carrying cells can be attacked and neutralized through co-regimen ISG15. This can be followed by ART to eliminate recurring viral replication and achieve eventual prevention of pathogen resurgence for complete viral clearance in HIV-infected patients *in vivo*.

## 10 Discussion

Cell–cell contacts between infected activated CD4^+^ cells and uninfected naïve resting or early differentiation CD4^+^CD25^−^, CD34^+^CD38^−^, or CD133^+^CD45^−^ expressing cells may allow (the passage of) HIV to become latent in these yet-to-be activated naïve or resting cells. Such virus entry is dependent on the permissive cell surface, intracellular and extracellular LFA1 conformational changes (Figures 1–3). Viral replication in the activated naïve cells may be required for IFN-γ secretion to elicit innate immunity such that the secreted CTL/NK effector vesicles can target and eliminate the infected cells. Here, the exogenously replenished ISG15 can kill the latency-reversed virus-harboring cells through effector-cell-mediated enhanced efficiency (Table 1). In the event of weakened or exhausted ISG15 potency, renewed viral replication in the LRA-reactivated cells can occur, including reinfections of the neighboring cells. Hence, periodically administered steady ISG15 levels may prevent reattachment of the latency-reversed virus to the LFA1 integrins of neighboring naïve or resting cells to reestablish latency via cell–cell contacts in the stromal microenvironment *in vivo*. Concomitant and coordinated PKC-prodrug activation of the latently infected cells that upregulate or become positive for CD69, together with exogenous recombinant ISG15 and alternating with ART may aid in efficient elimination of the infected cells that are conducive to renewal of replicative virus particles. This is indicated by the cessation of any positive reoccurrence of virus detection to achieve complete virus clearance in patients with HIV/AIDS.

The “chicken-and-egg” issue in the initial HIV1 infection of resting or activated CD4^+^ cells in humans *in vivo* may be considered herein. One can question whether LFA1 of the resting CD4^+^CD25^−^ cells preempts the activated CD4^+^CD25^+^ cells to receive this virus at the very first contact *in vivo* in the absence of an activated–resting intercellular contact since LFA1 also provides an entry mode for the virus. The viral entry via CD4 into the yet-to-be activated resting T-cells can reportedly lead to productive infection with replication cycles with the caveat that functional P-TEFb expression is a requirement in these CD4^+^CD25^−^ cells (Wietgrefe et al., 2023).

HIV latency most likely occurs via LFA1 in the CD34^+^/CD133^+^ early progenitor stem cells (that are yet to acquire a mature CD4 T-cell phenotype) when naïve CD34^+^/CD133^+^ cells come into contact with activated T-cells carrying the replicating virus, when both cell types are in contact within the stromal niches *in vivo*. Direct LFA1-mediated viral entry into these primitive or early yet-to-mature stem-progenitor cells is even less plausible because of the bent-closed LFA1 conformation, where access to the virus occurs even in the absence of such cell–cell contacts between *two* primitive-differentiation-stage progenitor cells. Similar to the adult stem cells, it may be possible for the stem-progenitor cells to engage in global suppression of transcription (Freter et al., 2010). This may occur in the progenitor cells at a more primitive stage than a mature differentiation stage while still lacking the CD4 phenotypic cell subpopulation. The bent-closed LFA1 conformation in the primitive cells could at least partially open up in the run-up to mature cells to allow HIV entry into the intrinsically non-phenotypic lineage-committed or mature progenitors where LFA1 acquires a virus-entry-supportive conformation.

HIV1 LRAs prostratin and bryostatin-1 have been reported to adversely affect the blood–brain barrier (BBB) (Dental et al., 2017). The colony stimulating factor-1 receptor (CSF1R) inhibitor, BLZ945, was found to mostly eradicate simian immunodeficiency virus infection of the non-human primate *rhesus macaque* brain CD163 and CD206 expressing perivascular macrophages (Bohannon et al., 2024), leaving the largely uninfected but required microglia preservation (Pasciuto et al., 2020) mostly intact. Moreover, since then, improved LRA-prodrugs are synthesized (Sloane et al., 2020) that are also useful for such patients suffering from HIV infection of their brains. Hence, additional drugs such as BLZ945 as necessary, be included as part of the ART as suggested (Table 1) to clear the presence and detection of otherwise systemic LRA-evasive virus particles from across the BBB. Such co-regimen with ART has potential applications for virus elimination systemically from different infected cells including macrophages.

In conclusion, although we can argue that excessive IFN-γ secretion in HIV infection is proinflammatory and undesirable, optimal levels are required to maintain antiviral responses for sustained efficacy of the effector cells (Table 1). Hence, in addition to and in conjunction with ISG15, periodic intermittent challenges with appropriate LRAs should be considered for complete clearance of the latent virus.

The question arises whether the addition of ISG15 in the combination ISG15 + LRA therapy can suffice or be effectively efficacious for permanent virus clearance in HIV-infected patients. In general, therapies for clinical conditions are invariably fraught with adverse side-effects. We suggest that clinical trials be conducted with HIV/AIDS patients to achieve post-ART-mediated minimal detectable virus loads *in vivo* and subsequent administration of ISG15 in combination with LRA-prodrug PKCMs alternating with ART. Appropriate recombinant ISG15 infusions are expected to maintain the innate immunity relatively free from exhaustion and weakening of CTL + NK effector cells and strengthen their responses. Thus, an intermittent periodic LRA/PKCM + ISG15 combination mediated intracellular release alternated with ART is suggested to prevent intercellular spread and renewal of the released latent virus replication. ART efficacy may also be synergized with ISG15-mediated enhanced dual-mode killing of the infected cells through ISG15-enhanced IFN-γ and perforin secretion by the vesicles released by the rejuvenated effector cells.

## Data Availability

The original contributions presented in this study are included in the article/supplementary material, and any further inquiries may be directed to the corresponding author.

## Abbreviations

IFN, interferon; ISG, interferon stimulated gene; LFA, lymphocyte function-associated antigen; ICAM, intercellular adhesion molecule; ESPC, endothelial stem-progenitor cells; HSPC, hematopoietic stem-progenitor cells; ART, antiretroviral therapy; LRA, latency-reversing agent; PKCM, protein kinase C modulator; CTL, cytotoxic T-lymphocytes; NK cells, natural killer cells; ACE2, angiotensin-converting enzyme-2; P-TEFb, positive transcription elongation factor b; HIV, human immunodeficiency virus; BBB, blood–brain barrier.
